# Advances in Our Understanding of the Mechanism of Action of Drugs (including Traditional Chinese Medicines) for the Intervention and Treatment of Osteoporosis

**DOI:** 10.3389/fphar.2022.938447

**Published:** 2022-06-14

**Authors:** Junjie Lu, Desheng Hu, Chen Ma, Bo Shuai

**Affiliations:** Department of Integrated Traditional Chinese and Western Medicine, Union Hospital, Tongji Medical College, Huazhong University of Science and Technology, Wuhan, China

**Keywords:** bone, osteoporosis, fracture, osteoblast, osteoclast, antiresorptive agent, pharmacological mechanism

## Abstract

Osteoporosis (OP) is known as a silent disease in which the loss of bone mass and bone density does not cause obvious symptoms, resulting in insufficient treatment and preventive measures. The losses of bone mass and bone density become more severe over time and an only small percentage of patients are diagnosed when OP-related fractures occur. The high disability and mortality rates of OP-related fractures cause great psychological and physical damage and impose a heavy economic burden on individuals and society. Therefore, early intervention and treatment must be emphasized to achieve the overall goal of reducing the fracture risk. Anti-OP drugs are currently divided into three classes: antiresorptive agents, anabolic agents, and drugs with other mechanisms. In this review, research progress related to common anti-OP drugs in these three classes as well as targeted therapies is summarized to help researchers and clinicians understand their mechanisms of action and to promote pharmacological research and novel drug development.

## Introduction

**FIGURE 1:**
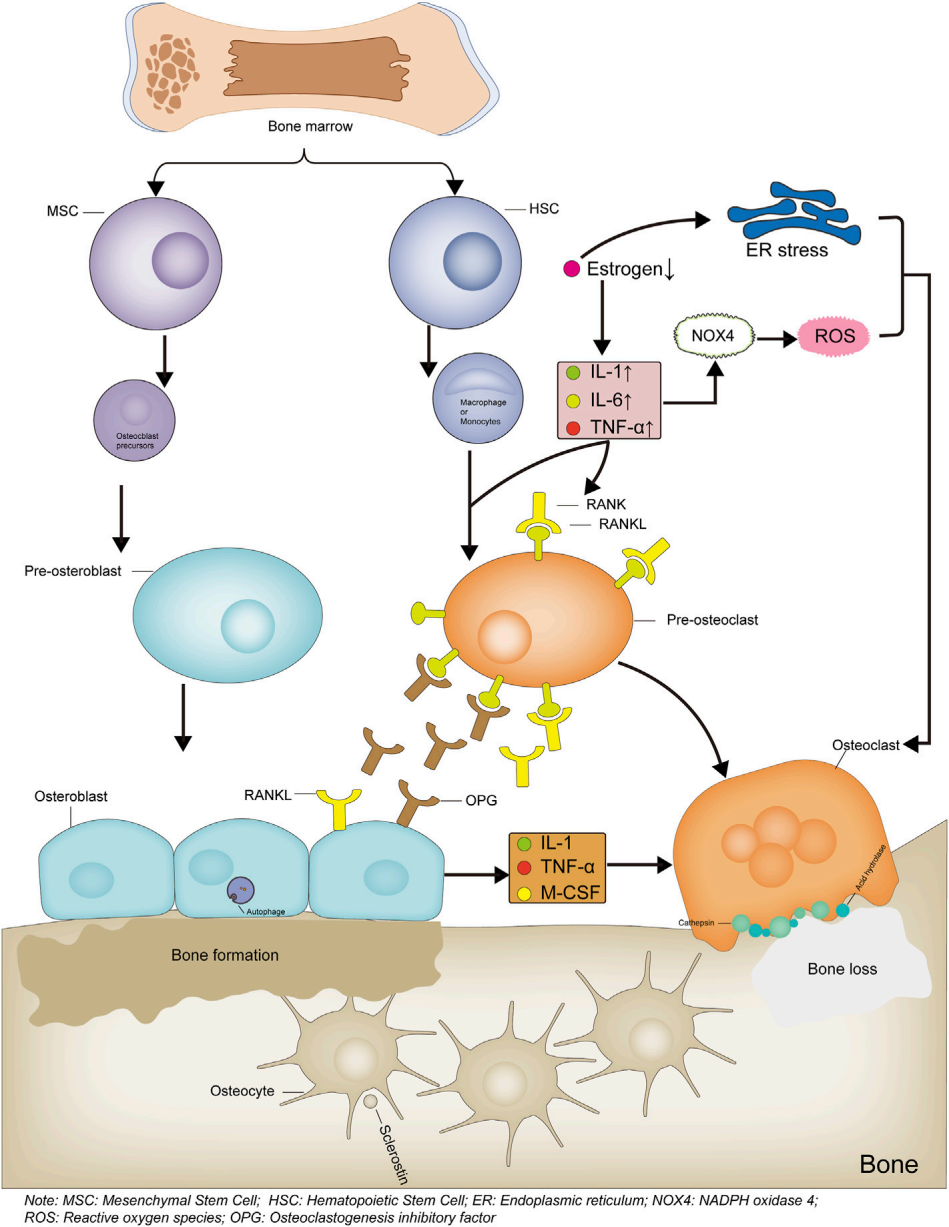
Mechanism diagram of bone formation and loss. Bone marrow-derived hematopoietic stem cells differentiate into monocytes or macrophages, which then differentiate into osteoclast precursor cells, then differentiate into osteoclasts and participate in bone loss. When estrogen levels are reduced or inflammatory factors are activated, the RANKL/RANK signaling pathway is activated, leading to increased osteoclast differentiation. The increased ROS levels or endoplasmic reticulum stress also leads to increased osteoclast differentiation. Bone marrow-derived mesenchymal stem cells differentiate into osteoblast precursor cells, which then differentiate into osteoblasts and participate in bone formation. The Osteoblasts produce OPG, competitively bind RANKL and inhibit osteoclast differentiation, thereby reducing osteoclast production. Bone formation and bone loss are in dynamic balance to maintain bone integrity and normal bone structure and function. Bone formation and bone loss are in dynamic balance to maintain skeletal integrity, normal bone structure and function.

The World Population Prospects 2019 published by the United Nations states that the proportion of people over 65 years of age is expected to rise to 16% globally by 2050, and the incidence of osteoporosis (OP) is increasing exponentially with this rapid increase in aging. In addition to age-related OP, other factors, such as the inappropriate use of glucocorticoids, alcohol abuse, and malnutrition, are common causes of secondary OP. Fragility fractures due to OP result in long-term pain and impaired mobility, leading to physical and psychological damage and causing a heavy burden on society. Up to one-quarter of patients with hip fractures die within 1 year. The high rates of disability and mortality from OP-related fractures cannot be ignored and thus must be treated aggressively (Hoogendijk et al., 2019). Bone integrity is maintained by the processes of bone resorption and osteogenesis, which involve osteocytes, osteoblasts, and osteoclasts. A positive balance between osteogenesis and bone resorption maintains normal bone structure and function, while a negative balance results in bone loss (Figure 1). This balance is determined by the production and function of osteoblasts and osteoclasts, which are regulated by multiple factors (T. L. Yang et al., 2020).

**FIGURE 2:**
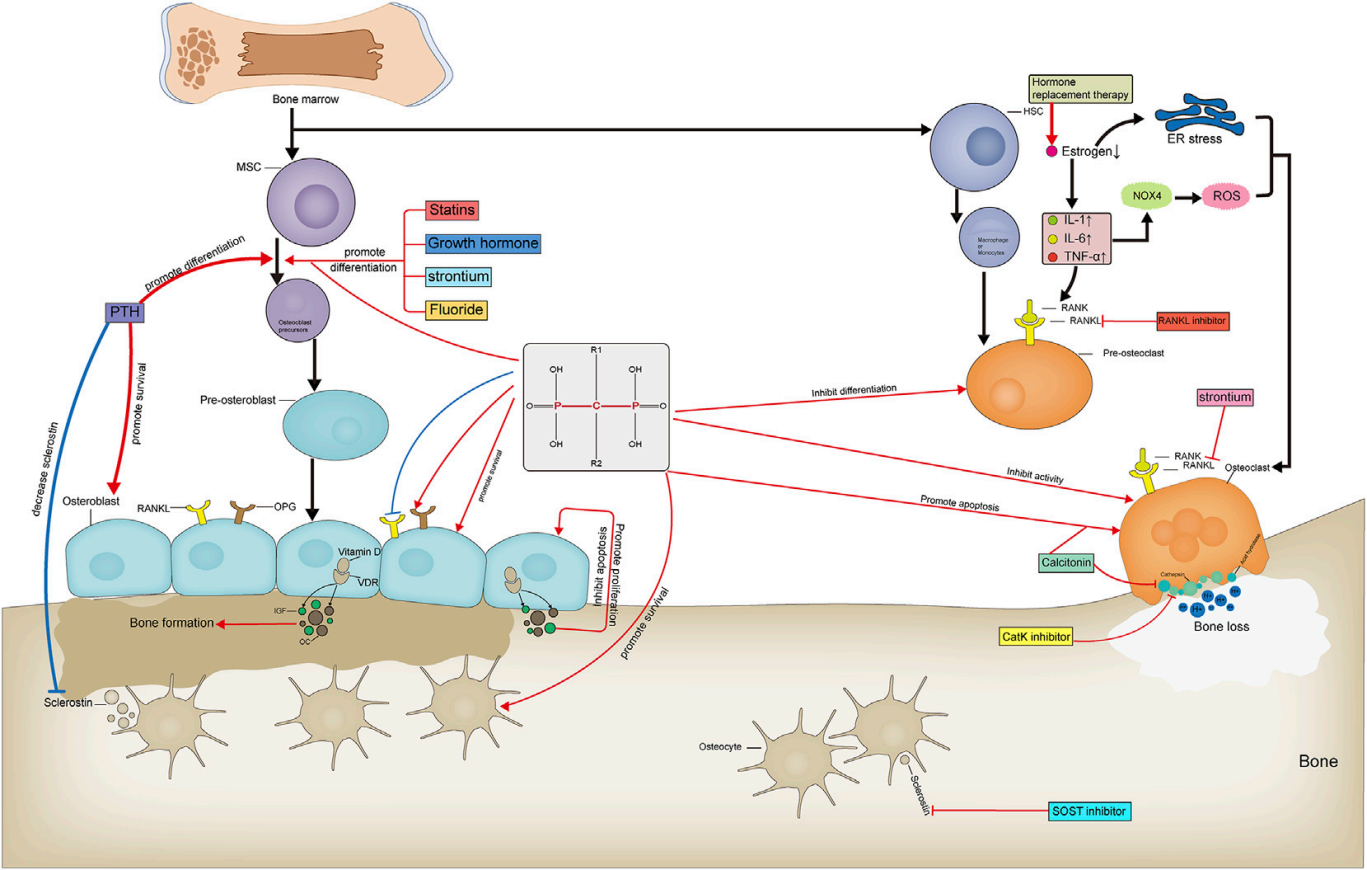
Targets and mechanisms of anti-osteoporosis drug action with different mechanisms. Pharmacological mechanism of anti-osteoporosis drugs affecting bone formation and bone resorption.

Currently, anti-OP drugs are divided into three classes: antiresorptive agents, anabolic agents, and drugs with other mechanisms of action. Commonly used antiresorptive agents include bisphosphonates, hormone replacement therapy, and calcitonin. Anabolic agents include parathyroid hormone and parathyroid hormone-related protein analogs, fluoride, growth hormone, and statins. Other drugs include strontium salts, vitamin D, vitamin K, and traditional Chinese medicines (TCM). Receptor Activator of Nuclear Factor-κB Ligand (RANKL) inhibitors, SOST inhibitors, and cathepsin K inhibitors are not included within the three major classes as targeted anti-OP agents (Table1). We systematically review common anti-OP drugs in each of these classes and discuss their molecular mechanisms of action to guide their standardized clinical use, pharmacological research, and the development of novel targeted drugs. Figure 2: Targets and mechanisms of anti-osteoporosis drug action with different mechanisms.

**TABLE 1:** Some anti-osteoporpsis durgs currently in use.

Types	Drugs	Administration pathways	Dosage for treatment	Brief function mechanism
Bisphosphonates	Alendronate	p.o.	10 mg Q.d or 70 mg Qw	Inhibit bone conversion;
	Zoledronic acid	p.o.	5 mg/year	
	Risedronate	p.o.	5 mg Q.d or 35 mg Qw	Inhibit osteoclast recruitment on the bone surface;
	Ibandronate	p.o.	2 mg/once/3 month	
	Etidronate disodium	p.o.	0.2 g Bid	Induce osteoclast apoptosis;
	Clodronate disodium	p.o.	0.4 g Bid or 0.8 g Q.d	
Hormone replacement therapy	Estrogen/progestogen	p.o.	customized	the inhibition of RANKL / rank activation;
	Raloxifene	p.o.	60 mg Q.d	the inhibition of oxidative stress;
				the inhibition of local inflammation
Calcitonin	Elcatonin	i.m.	20 U Qw or 10 U/twice/week	Inhibit osteoclast activity;
	Salmon calcitonin	i.m./Inhal	i.m.: 50 or 100 U Q.d/Inhal: 200 U Q.d	Inhibit acid hydrolase release;
				Reduce osteoclast adhesion on bone surface;
				Reduce blood calcium concentration
Parathyroid hormone and its analogs	Abaloptide	i.h.	80 ug Q.d	Regulation of serum calcium and phosphorus ion concentration
	Teriparatide	i.h.	20 ug Q.d	
Vitamin D and its analogs	α-calciferol	p.o.	0.25–1 ug Q.d	Regulation of calcium and phosphorus reabsorption
	Calcitriol	p.o.	0.25–0.5 ug Bid or 0.5 ug Q.d	
Strontium	Strontium ranelate	p.o.	2 g Q.d	Regulate Runx2;
				Activate non-classical Wnt pathway;
				Inhibit RANKL/RANK-induced osteoclastic differentiation
Vitamin K	Menatetrenone	p.o.	15 mg Tid	Enhanced cartilage protection
Traditional Chinese Medicine	customized	p.o.	customized	Multiple targets, multiple pathways and multiple mechanisms
Targeted agents for anti-osteroporosis	RANKL inhibitor-Denosumab	i.h.	60 mg/once/180 d	Specific targeting NF-κB receptor activator ligand
	Cat-K inhibitor-Odanacatib	p.o.	50 mg Qw	Selective inhibitor of Cathepsin K

### Antiresorptive Agents

#### Bisphosphonates

Bisphosphonates are the most widely used drugs for the treatment of OP. They effectively reduce the risk of vertebral, non-vertebral, and hip fractures (Burr & Russell, 2011). The mainstream view is that bisphosphonates inhibit bone resorption by three principal mechanisms: 1) inhibition of bone turnover, 2) direct inhibition of osteoclasts and indirect promotion of osteoblast recruitment to the bone surface (Sato et al., 1991), and 3) induction of apoptosis in osteoclasts (Plotkin et al., 1999) (Plotkin, Aguirre, Kousteni, Manolagas, & Bellido, 2005) and inhibition of apoptosis in osteoblasts (Plotkin et al., 2008). The mechanism by which bisphosphonates inhibit bone resorption is related to their specific chemical structure. Bisphosphonates are derivatives of inorganic pyrophosphates, in which the central oxygen atom of the inorganic pyrophosphate molecule is replaced with a carbon atom, thus forming a P-C-P bond (Fleisch, Russell, & Francis, 1969). The presence of this chemical bond confers a strong affinity with calcium phosphate, leading to the inhibition of normal and ectopic mineralization. The structure of the two side chains: R1 and R2 on the central carbon atom determines the potency of the particular bisphosphonate; alendronate has a four-carbon atom backbone and exhibits the best activity, while zoledronate is a cyclic bisphosphonate with a nitrogen ring and exhibits the highest relative potency (Luckman et al., 1998; E. van Beek, Pieterman, Cohen, Löwik, & Papapoulos, 1999a, 1999b) (Tafaro & Napoli, 2021). Simple bisphosphonates that closely resemble inorganic pyrophosphonates bind to newly formed adenosine triphosphate (ATP) molecules via class II aminoacyl-tRNA synthetases, and these analogs accumulate within osteoclasts, thereby inducing apoptosis (Pelorgeas, Martin, & Satre, 1992; Sillero et al., 2009). A more clinically effective mechanism underlying the inhibition of osteoclast bone resorption is the inhibition of farnesyl pyrophosphate (FPP) synthase and/or isopentenyl pyrophosphate (IPP) isomerase activity using nitrogen-containing bisphosphonates. The inhibition of these essential enzymes in the mevalonate pathway decreases FPP and geranylgeranyl pyrophosphate (GGPP), resulting in reduced prenylation of the Rac, Ras, and Rho signal transduction proteins, in turn leading to a loss of osteoblast function (Dunford et al., 2001; E. R.; Van Beek, Löwik, & Papapoulos, 2002). These proteins may be intracellular targets of nitrogen-containing bisphosphonates (Kavanagh et al., 2006).

In addition to inhibiting osteoclast function and inducing osteoclast apoptosis, some studies have also shown that bisphosphonates have effects on osteoblast and osteocyte function. Bisphosphonates directly promote osteoblast proliferation and increase osteoblast differentiation. Additionally, bisphosphonates can directly promote osteoblast survival to exert anti-apoptotic effects (Igarashi, Hirafuji, Adachi, Shinoda, & Mitani, 1997; Giuliani et al., 1998). This effect is dependent on the ability of bisphosphonates to rapidly induce ERK phosphorylation, and when ERK activation is inhibited, the anti-apoptotic effect of bisphosphonates is eliminated. Furthermore, bisphosphonates can also reduce RANKL expression in osteoblasts and increase osteoprotegerin (OPG) expression to exert anti-osteoporosis effects (Viereck et al., 2002; Pan et al., 2004). Similarly, bisphosphonates can maintain osteocyte activity and inhibit glucocorticoid-induced osteocyte apoptosis (Plotkin et al., 1999; Plotkin et al., 2005). Bisphosphonates also have a modulatory effect on bone marrow mesenchymal stem cells. Bisphosphonates could enhance the proliferation of bone marrow stromal cells and promoting osteogenic differentiation. Bisphosphonates promote the expression of the osteogenic genes *alkaline phosphatase (ALP)*, *bone morphogenetic protein-2 (BMP-2)* and *osteocalcin (OC)* in marrow mesenchymal stem cells (MSCs) (Ribeiro et al., 2014) and directly enhance osteoblast formation and subsequent mineral deposition, which may be another mechanism by which they exert anti-osteoporosis (Lindtner et al., 2014). MSC-like cells isolated from peripheral blood of osteoporotic patients with intervention with bisphosphonates showed significantly increased expression levels of the osteoblast marker gene *Runt-associated transcription factor 2 (RUNX2)*, *BMP-2* and bone turnover markers C-terminal telopeptide (CTX), pre-collagen type 1 N-terminal pro-peptide (P1NP) and bone alkaline phosphatase (bALP) levels were significantly reduced (Dalle Carbonare et al., 2017; von Knoch et al., 2005).

Due to the effectiveness, safety, and affordability of bisphosphonates in preventing fractures, they have been used as the most common drug for postmenopausal osteoporosis and as a first-line agent (Black & Rosen, 2016; Ensrud & Crandall, 2019). Patients with postmenopausal osteoporosis who received annual intravenous infusions of zoledronic acid had a 70% reduction in the risk of lumbar vertebrae fracture and a 41% reduction in hip fracture over 3 years, with varying degrees of reduction in other fractures (Black et al., 2007). Treatment of postmenopausal women with osteopenia without osteoporosis with zoledronic acid also reduced the incidence of non-lumbar vertebrae or lumbar vertebrae fragility fractures, so zoledronic acid could be used to prevent fractures in older women with osteopenia. Zoledronic acid also has a better preventive effect on osteoporosis and secondary fractures caused by inappropriate glucocorticoid use, which increases lumbar spine bone mineral density (BMD) (D. M. Reid et al., 2009). After discontinuation of the drug, the effect of bisphosphonates gradually disappeared, and a decrease in BMD and a gradual increase in biochemical markers of bone turnover occurred within 5 years (Bone et al., 2004; Black et al., 2006). In the use of bisphosphonates, attention should be paid to their use characteristics and alert to the occurrence of adverse reactions. Differences exist between the injectable and oral forms of bisphosphonates. Gastrointestinal dysfunction and gastrointestinal reactions occur in about 20% of patients taking oral bisphosphonates, and transient acute allergy-like symptoms with fever, musculoskeletal pain, and flu-like symptoms are present in 20–30% of patients when the injectable form is first used; most of these symptoms resolve within 3 days and can be effectively prevented with acetaminophen or ibuprofen for a short time after administration. In addition, intravenous administration of bisphosphonates can lead to renal impairment as evidenced by elevated blood creatinine levels or rare acute renal failure, and is therefore contraindicated in patients with creatinine clearance <35 ml/min. Appropriate rehydration should be administered prior to the use of intravenous dosage forms, and single doses and infusion rates should be controlled during use. Osteonecrosis of the jaw is a rare but serious complication of bisphosphonate use, but this is often seen in patients treated with bisphosphonates for myeloma or other bone tumors at doses more than 10 times the dose of osteoporosis treatment. (Maraka & Kennel, 2015; Eastell et al., 2016; Compston, McClung, & Leslie, 2019).

#### Hormone Replacement Therapy

Women after menopause are over 50% more likely than men of the same age to experience OP-related fractures, with estrogen deficiency being the primary cause. Estrogen deficiency leads to increased bone turnover, with bone resorption increasing by 90% after menopause and osteogenesis increasing by only 45% (Garnero, Sornay-Rendu, Chapuy, & Delmas, 1996); this high level of bone turnover leads to bone loss during the bone remodeling cycle. Receptors with high affinity for estrogen are present in osteoblasts, osteoclasts, and osteocytes. An estrogen deficiency directly increases osteoclast formation and bone matrix degradation and decreases osteogenesis-related gene expression (Schiavi, Fodera, Brennan, & McNamara, 2021). It also decreases osteoblast autophagy, leading to increased osteoblast apoptosis (Schiavi et al., 2021) and the activation of endoplasmic reticulum stress, which results in reduced osteoblast formation and reduced matrix mineralization (Gavali et al., 2019; H.; Li et al., 2018). In addition, an estrogen deficiency induces increased activity of the osteoclastic cytokines IL-1, IL-6, and TNF-α(Pacifici et al., 1991; Kimble et al., 1994; Ammann et al., 1997). T cell activation also releases large amounts of TNF-α(Roggia et al., 2001), and the synergistic effects of these pathways lead to the activation of NF-κB (RANK) and NF-κB ligand (RANKL). This pathway induces osteoblasts to express NADPH oxidase 4 (NOX4), leading to increased levels of reactive oxygen species and subsequent increases in osteoclastogenesis (Goettsch et al., 2013). This leads to the formation of a pro-inflammatory environment locally in bone and exacerbates tissue damage. Theoretically, OP can be effectively intervened and treated with estrogen replacement therapy. The bone mineral density was increased and fracture risk was decreased in postmenopausal women after 3 years of estrogen use (Cauley et al., 2003). However, clinical estrogen supplementation for postmenopausal OP must be evaluated with caution owing to the potential for adverse cardiovascular and cerebrovascular events, thromboembolic disease, and an increased incidence of breast cancer in women (G. L. Anderson et al., 2004; LaCroix et al., 2011; Rozenberg, Vandromme, & Antoine, 2013). Thus, the prudent evaluation and rational short-term use of estrogen should be prioritized over long-term maintenance therapy.

One strategy that can be used as a long-term maintenance of estrogen replacement therapy is the use of selective estrogen receptor modulators (SERMs). SERM is different from endogenous estrogen in pharmacological action. It plays different roles in different tissues by binding with estrogen receptor and produces estrogen agonist effect on bone. Therefore, it can reduce bone resorption and increase bone mineral density (BMD) in postmenopausal women. In addition, the extraosseous effect of SERM is to regulate cholesterol metabolism and reduce the levels of total cholesterol and low-density lipoprotein cholesterol. The reduction of cholesterol level will lead to the nuclear condensation of osteoclasts, reduce the activity of osteoclasts and induce the activation of Caspase-3, so as to induce osteoclast apoptosis (Luegmayr et al., 2004), which may be similar to the mechanism of statins potentially reducing the risk of fracture. The only SERM recommended by JAMA for anti-osteoporosis treatment in 2019 is raloxifene (I. R. Reid & Billington, 2022). Among postmenopausal women with osteoporosis, raloxifene can increase the bone mineral density of spine and femoral neck and reduce the risk of vertebral fracture (Delmas et al., 1997; Walsh et al., 1998). While reducing the risk of fracture, raloxifene can also bring more clinical benefits. Raloxifene can reduce the concentration of serum total cholesterol and low-density lipoprotein cholesterol, and it can reduce the incidence rate of breast cancer in postmenopausal women with high levels of estradiol. Although it brings so many clinical benefits, its clinical application also has some problems. For example, it is only suitable for women and has poor effect in reducing non vertebral fractures. There is a significant increase in venous thrombosis and fatal stroke risk caused by the use of raloxifene, as well as the problem of intolerable vasomotor stability syndrome (hot flashes), which require clinicians to give comprehensive and careful consideration when using the drug (Ettinger et al., 1999; Cummings et al., 2002; Goldstein et al., 2009).

#### Calcitonin

Calcitonin is a naturally occurring single-chain polypeptide consisting of 32 amino acids and a 7-amino-acid ring structure (Andreotti et al., 2006). Calcitonin has two types of receptors: calcitonin receptor (CTR) and calcitonin receptor-like receptor (CLR). Calcitonin inhibits osteoclast function by binding to specific receptors (Nicholson, Moseley, Sexton, Mendelsohn, & Martin, 1986). The NH_2_-terminal loop structure is a key structural domain for receptor activation (Barwell et al., 2012). Calcitonin is present in many species; in humans, calcitonin is secreted by parafollicular cells of the thyroid (C cells) and acts on bones. Calcitonin has a direct inhibitory effect on osteoclast activity (Chambers & Magnus, 1982). Calcitonin exerts anti-OP effects by preventing osteoclasts from secreting acid hydrolases to degrade and resorb bone (Horne, Shyu, Chakraborty, & Baron, 1994). Calcitonin also directly causes osteoclasts to detach from the resorption lacuna and reduces the number of osteoclasts adhering to the bone (Holtrop, Raisz, & Simmons, 1974; Delling, Schulz, & Ziegler, 1977). The extent of hypocalcemia is positively correlated with the rate of bone resorption, and calcitonin antagonizes the increase in the blood calcium concentration by inhibiting the reabsorption of calcium and phosphorus ions by the renal tubules, thereby reducing the rate of bone resorption (Copp & Cheney, 1962; Hedlund, Hulth, & Johnell, 1983). Unlike other anti-OP drugs, calcitonin has the unique advantage of having analgesic effects and can relieve acute and chronic pain caused by OP-related vertebral compression fractures (Lyritis et al., 1997; Knopp-Sihota, Newburn-Cook, Homik, Cummings, & Voaklander, 2012; Terashima et al., 2019); the mechanism underlying this effect this remains unclear (Kaneb, Berardino, Hanukaai, Rooney, & Kaye, 2021). Previous studies have shown that calcitonin may exert analgesic effects by the modulation of the central nervous system as well as neurotransmitter release and neurophysiology (Pecile, Ferri, Braga, & Olgiati, 1975; Yamamoto, Tachikawa, & Maeno, 1981; Laurian et al., 1986). Although the mechanism of action remains unclear, the analgesic effect of calcitonin has been confirmed. Structural differences in calcitonin among species correspond to large differences in its affinity for calcitonin receptors. Eel calcitonin derivatives in which NH_2_-terminal amino groups are replaced with hydrogen atoms and disulfide bonds with ethylene bonds have high stability with no differences in biological activity. Salmon calcitonin, the most widely used peptide in clinical practice (Chesnut et al., 2000), has high affinity and biological potency in humans and a long half-life due to its high sequence homology with human calcitonin. The specific properties of the calcitonin peptide limit its modes of administration. It was previously administered as a parenteral injection but is now available as an intranasal formulation, with improved convenience and tolerability (Reginster & Franchimont, 1985). The development of new dosage forms of calcitonin to improve its bioavailability is a focus of ongoing research, including oral and inhaled forms as well as allosteric calcitonin receptor activator dosage forms (Watkins, Rathbone, Barwell, Hay, & Poyner, 2013).

### Anabolic Agents

#### Parathyroid Hormone and Parathyroid Hormone-Related Protein Analogs

Similar to calcitonin, PTH is a peptide consisting of 84 amino acids. PTH and its related protein analogs also act by binding to PTH 1 receptor (PTH1R), a class B G protein-coupled receptor with an important role in maintaining calcium ion homeostasis. Functionally, PTH and its related protein analogs function with calcitonin in the regulation of calcium and phosphorus ion metabolism in the blood and bone cell activity (Beutner & Munson, 1960; Felsenfeld, Levine, & Rodriguez, 2015) (Raisz, 1963; Bingham, Brazell, & Owen, 1969). The N-terminal sequence of PTH is highly conserved in many mammals; accordingly, the main biological activities of PTH are mediated by the binding of the N-terminal to PTH1R (Martin et al., 1983), while the C-terminal region is mainly involved in the regulation of bone resorption and serum calcium ions. In addition, mutations in the C-terminal sequence affects its affinity to the receptor and its own metabolic degradation rate (Potts et al., 1971). Animal studies have shown that the continuous administration of PTH leads to bone loss (Cosman, Shen, Herrington, & Lindsay, 1991), whereas intermittent administration leads to increased osteoblast numbers and osteogenesis (Frolik et al., 2003). Currently, JAMA lists two PTH analogs for the treatment of OP: teriparatide and abaloparatide (I. R. Reid & Billington, 2022). Teriparatide induces bone formation by stimulating the proliferation and activity of osteoblasts. This stimulation peaks at 6–12 months and decreases thereafter, even after a second course of treatment administered 1 year after discontinuation. Abaloparatide binds highly selectively to the R0 and RG conformations of PTH1R, resulting in a shorter cAMP response, and exhibits greater osteogenic effects than those of teriparatide (Hattersley, Dean, Corbin, Bahar, & Gardella, 2016). Toxicological studies of PTH and its related protein analogs in animals have shown that the lifetime use of PTH and its related protein analogs in excess of equivalent human doses of PTH results in an increased risk of osteosclerosis and osteosarcoma (Sato et al., 2002; Vahle et al., 2004). However, based on the stimulatory effects of PTH and its related protein analogs described above, teriparatide and abaloparatide are not used for the long-term treatment of OP.

#### Fluoride

Fluorine is an essential trace element in the human body and is particularly important for bone growth, development, and calcification. Fluorine promotes osteoblast proliferation and activation by stimulating the differentiation of mesenchymal stem cells into osteoblasts and inhibiting acid phosphatase activity in osteoblasts, leading to increased osteogenesis (Hall, 1987; Lau, Farley, Freeman, & Baylink, 1989). However, caution must be exercised in the use of fluoride to treat OP, as excessive fluorine intake can lead to decreased bone strength and toughness, increased insulin resistance, elevated inflammatory factor levels, and disturbances of lipid metabolism, all of which can lead to adverse events (de Cássia Alves Nunes et al., 2016; Pereira et al., 2017). In addition, the development of gastrointestinal disturbances in a large proportion of patients (34%) due to the use of fluoride and a variety of adverse effects, such as fluorosis, have limited its application (Franke, 1988).

#### Growth Hormone

A growth hormone deficiency in adults leads to a decreased bone mineral density; however, it is not clear whether growth hormone supplementation is beneficial for aging-related bone loss. Clinical studies have shown that growth hormone activates osteoblasts and stimulates bone remodeling in older men, women, and postmenopausal patients with OP but has no long-term effects on the spine and proximal femur bone density (Brixen, Nielsen, Mosekilde, & Flyvbjerg, 1990; Rudman et al., 1990; Ghiron et al., 1995). A meta-analysis has shown that the incidence of adverse events in patients treated with growth hormone was 24.8 ± 28.6% compared to 6.1 ± 7.8% in the control group. Both meta-analyses have shown that the most common adverse effects of growth hormone use were pain and edema due to fluid retention and carpal tunnel syndrome (Atkinson, Moyer, Yacoub, Coughlin, & Birmingham, 2017; Barake et al., 2018).

#### Statins

Statins are frequently used and have been found to regulate lipids and to reduce the incidence of fractures. Some studies have shown that the statin compactin promotes osteoblast differentiation and bone nodule formation (Phillips, Belmonte, Vernochet, Ailhaud, & Dani, 2001), which may be associated with the activation of the Ras/Smad/Erk/BMP-2 pathway (P. Y. Chen et al., 2010). However, clinical observations have yielded mixed findings. For example, comparative study published in the Lancet in 2020 showed that 13 or more doses of statins reduced the risk of pathological bone fracture in women over 60 years of age (Chan et al., 2000). A systematic evaluation and meta-analysis of the efficacy of statins for OP by Zou *et al.* revealed that statins reduced the overall fracture risk, increased hip BMD, and increased osteocalcin expression, with no significant effects on the femoral neck BMD and bone-specific alkaline phosphatase (BALP) and serum collagen type I telopeptide (S-CTX). In addition, statins had greater therapeutic effects against OP in male patients than in female patients (An et al., 2017). However, other clinical studies have reported the opposite results. In a clinical observation of 93,716 postmenopausal women aged 50–79 years, statin use did not improve the BMD or fracture risk (LaCroix et al., 2003). Therefore, definitive conclusions cannot be drawn about the relationship between statin use and OP and more molecular biological and clinical studies are needed.

### Drugs With Other Mechanisms of Action

#### Vitamin D

Vitamin D is obtained through dietary sources and exposure to sunlight. Upon exposure to sunlight (especially wavelengths of 280–320 nm), 7-dehydrocholesterol is converted to a previtamin, which is then slowly isomerized to vitamin D_3_, and ergosterol is converted to vitamin D_2_. Vitamin D is metabolized in three major steps: 25-hydroxylation, 1α-hydroxylation, and 24-hydroxylation (Bikle, 2014). Vitamin D is first hydroxylated to 25-hydroxyvitamin D by CYP27A1 and CYP2R1, with CYP27A1 acting only on vitamin D_2_ and CYP2R1 acting on both vitamins D_2_ and D_3_. Other enzymes, such as CYP3A4, which is primarily involved in drug metabolism, also have 25-hydroxylation activity; however, CYP27A1 and CYP2R1 are the primary enzymes involved in the 25-hydroxylation process. 25-Hydroxyvitamin D produced in the liver undergoes 1α-hydroxylation catalyzed by CYP27B1 (1α-hydroxylase) into 1α-25-dihydroxyvitamin D, which in turn undergoes 23-hydroxylation catalyzed by CYP24A1 (23-hydroxylase) into the biologically active 1, 25–26,23 lactone. It also undergoes 24-hydroxylation catalyzed by CYP24A1 (24-hydroxylase), forming 1,24,25-trihydroxyvitamin D, which binds to the vitamin D receptor and performs a variety of functions, including calcium absorption, intestinal phosphate absorption, bone calcium mobilization, and renal calcium reabsorption (Meyer, Goetsch, & Pike, 2010; Jones, Prosser, & Kaufmann, 2012). Of these steps, 25-hydroxylation occurs primarily in the liver, while 1α-hydroxylation and 24-hydroxylation occur primarily in the kidney. 25-Hydroxylase in the liver is regulated by several factors; therefore, the double deletion of CYP27A1 and CYP2R1 does not completely block 25-hydroxylation in the liver. Renal 1α-hydroxylase is primarily regulated by PTH, fibroblast growth factor 23 (FGF23), and 1α,25-dihydroxyvitamin D itself (Armbrecht et al., 1998; Bacchetta et al., 2013). PTH stimulation promotes CYP27B1 production, while FGF23 and high calcium (Ca) and phosphorus (P) inhibit CYP27B1 production; CYP24A1 is regulated in the opposite direction. 1, 25 (OH)_2_D_3_ also directly regulates its own synthesis by inhibiting PTH production, stimulating FGF23 production, and inducing CYP24A1 production.

Currently, the effect of vitamin D supplementation on the risk of fracture is controversial. Chapuy *et al.* reported a 43% reduction in the risk of hip fracture and a 32% reduction in the risk of non-vertebral fractures in 3,270 elderly French women who took 1,200 mg of calcium and 800 units of vitamin D_3_ daily for 3 years (Chapuy et al., 1992). However, many recent studies have questioned this finding, and a 2017 meta-analysis found that neither calcium, vitamin D, nor a combination of the two reduced the fracture risk in older adults, controlling for calcium and vitamin D dose, gender, and baseline serum 25-hydroxyvitamin D concentrations (Zhao, Zeng, Wang, & Liu, 2017). Similarly, a 2019 study found that even 3 years of treatment with high doses (10,000 IU) of vitamin D daily did not produce significant changes in radial or tibial bone strength (Burt et al., 2019). These findings suggest the importance of individualized vitamin D supplementation based on baseline 25-hydroxyvitamin D concentrations and patient conditions, such as exposure to outdoor light levels and differences in joint loading due to differences in body weights and activity levels, rather than the universal use of vitamin D to prevent OP and bone fracture across all populations. Several analyses have shown that adjusting serum 25-hydroxyvitamin D concentrations to 90–100 nmol/L (36–40 ng/ml) effectively reduces the fracture risk (Bischoff-Ferrari, Giovannucci, Willett, Dietrich, & Dawson-Hughes, 2006; Leidig-Bruckner et al., 2011; Bischoff-Ferrari, 2014).

A major cause of fracture in some elderly people is the increased risk of falls due to a lack of muscle strength and muscle fatigue due to a vitamin D deficiency, as skeletal muscles contain many vitamin D receptors (Dawson-Hughes, 2017). With increasing age, vitamin D receptor expression decreases (Bischoff-Ferrari et al., 2004), and if vitamin D is deficient, the risk of muscle fatigue may be exacerbated. Appropriate vitamin D supplementation is effective in improving muscle strength and movement speed, which can reduce the risk of falls in the elderly (Bouillon et al., 2019). This may explain the inconsistent results of clinical trials, which did not consider differences in muscle levels between samples.

#### Strontium Salts

Strontium is a trace element with similar physical and chemical properties to those of calcium ions, enabling it to partially replace their function (Kołodziejska, Stępień, & Kolmas, 2021). Strontium has a unique anti-OP mechanism that affects both bone resorption and osteogenesis (Peng et al., 2011). On one hand, it promotes the differentiation of bone marrow mesenchymal stem cells (BMSCs) into osteoblasts by regulating Runx2, a key transcription factor for osteoblast differentiation, and activating NFATc1/Maf via the canonical and non-canonical Wnt pathways (Cui et al., 2020). On the other hand, it inhibits the expression of RANKL and AP-1 by interacting with calcium-sensing receptor (CaSR) and antagonizes the NF-kB-mediated differentiation of bone marrow MSCs into osteoblasts (Brennan et al., 2009; Saidak, Haÿ, Marty, Barbara, & Marie, 2012). Strontium ranelate is one of a few drugs currently available for the treatment of postmenopausal OP. In 2013, the European Medicines Agency recommended the discontinuation of strontium ranelate because it increases the risk of cardiovascular disease and venous thrombosis and thus its risks outweigh its benefits. In addition, the FDA has not approved the drug for clinical use; therefore, it is not detailed further in this review.

#### Vitamin K

Vitamin K (VK) is a fat-soluble vitamin that has effects on bone in addition to activating prothrombin to promote coagulation (Tsugawa & Shiraki, 2020). VK is a coenzyme necessary for the synthesis of γ-carboxyglutamate and can facilitate the transition from inactive low carboxylation levels to fully functional active carboxylation levels. Skeletal malformations have been found in patients using the VK antagonist warfarin, suggesting that VK is associated with bone and bone metabolism (Palermo et al., 2017). A number of studies have shown that VK regulates bone metabolism via both VK-dependent and non-VK-dependent protein pathways. Since studies of non-VK-dependent protein pathways have focused on osteoarthritis, we focus here on the relationship between VK-dependent proteins and OP. There are six VK-dependent proteins in bone tissues: osteocalcin, matrix GLA protein (MGP), upper zone of growth plate and cartilage matrix-associated protein (UCMA), also known as GLA-rich protein (GRP), periostin, protein S, and growth arrest-specific 6 protein (Gas6) (Hauschka, Lian, Cole, & Gundberg, 1989; Shearer, 2000). These proteins regulate bone metabolism by promoting osteoblast differentiation and inhibiting osteoclastogenesis (Stock & Schett, 2021).

VK acts as a coenzyme for carboxylase to promote osteocalcin carboxylation levels and increase calcium ion and hydroxyapatite binding activity for hydroxyapatite crystal formation (Atkins, Welldon, Wijenayaka, Bonewald, & Findlay, 2009). The GLA residue-rich structure of MGP results in a high affinity for calcium ions and hydroxyapatite. UCMA and GRP have multiple functions, including a well-established protective effect on cartilage and the ability to bind to types II, I, and XI collagen with high affinity (Wallin, Schurgers, & Loeser, 2010; Theuwissen, Smit, & Vermeer, 2012). However, their roles in osteoblast differentiation are not clear. There is some evidence that UCMA/GRP inhibits osteoblast differentiation, while other studies have shown that UCMA/GRP overexpression in osteoblasts promotes osteogenic differentiation (Houtman et al., 2021). These results were based on transcript-level analyses, and differences in levels of γ-carboxylation and post-translational modification require further study. Relatively few studies have evaluated periostin, Gas6, and protein S and therefore they are not reviewed here for the sake of brevity. Some clinical studies of VK and OP have been reported, and a systematic review and meta-analysis has shown that patients using VK antagonists had a greater risk of fracture, but VK antagonists were not associated with BMD (Veronese et al., 2015). Clinical evidence for VK supplementation in patients with OP is lacking, and long-term follow-up studies are required to elucidate the short- and long-term effects of VK on bone.

#### Traditional Chinese Medicines

China has over 2000 years of rich history and experience in the use of herbal medicines. The use of TCM for the intervention and treatment of OP has been included in the latest Chinese clinical guidelines. However, most TCM use involves the personal experience of clinicians and their lineage, without specific indications, scope of use, documentation of adverse reactions and contraindications, modern pharmacological studies, and standardized clinical phase III trials. The complex composition and multi-pathway and multi-target mechanisms of TCM pose great difficulties for research. Research on modern pharmacological and molecular mechanisms of action of TCM is being promoted (Shuai, Shen, Zhu, & Zhou, 2015). We need to standardize their scientific application, elucidate their pharmacological effects and mechanisms, define their safety and toxic side effects, and accelerate the innovation of drug formulations and absorption methods.

Anti-OP components in TCM are diverse and include flavonoids, lignans, saponins, and iridoid glycosides (Sun et al., 2021). They function via multiple pathways, such as the Wnt/β-catenin, BMP/Smad, PI3K/AKT, MAPK, and RANKL/OPG pathways (Stock & Schett, 2021; Yang et al., 2020). Their therapeutic mechanisms are similar to those of the drugs described earlier and they can regulate bone turnover and exert other anti-OP effects via the signaling pathways described earlier (He et al., 2019). However, TCM has the characteristics of multi-components and mult-targets. *Psoralea corylifolia*.

**FIGURE 3:**
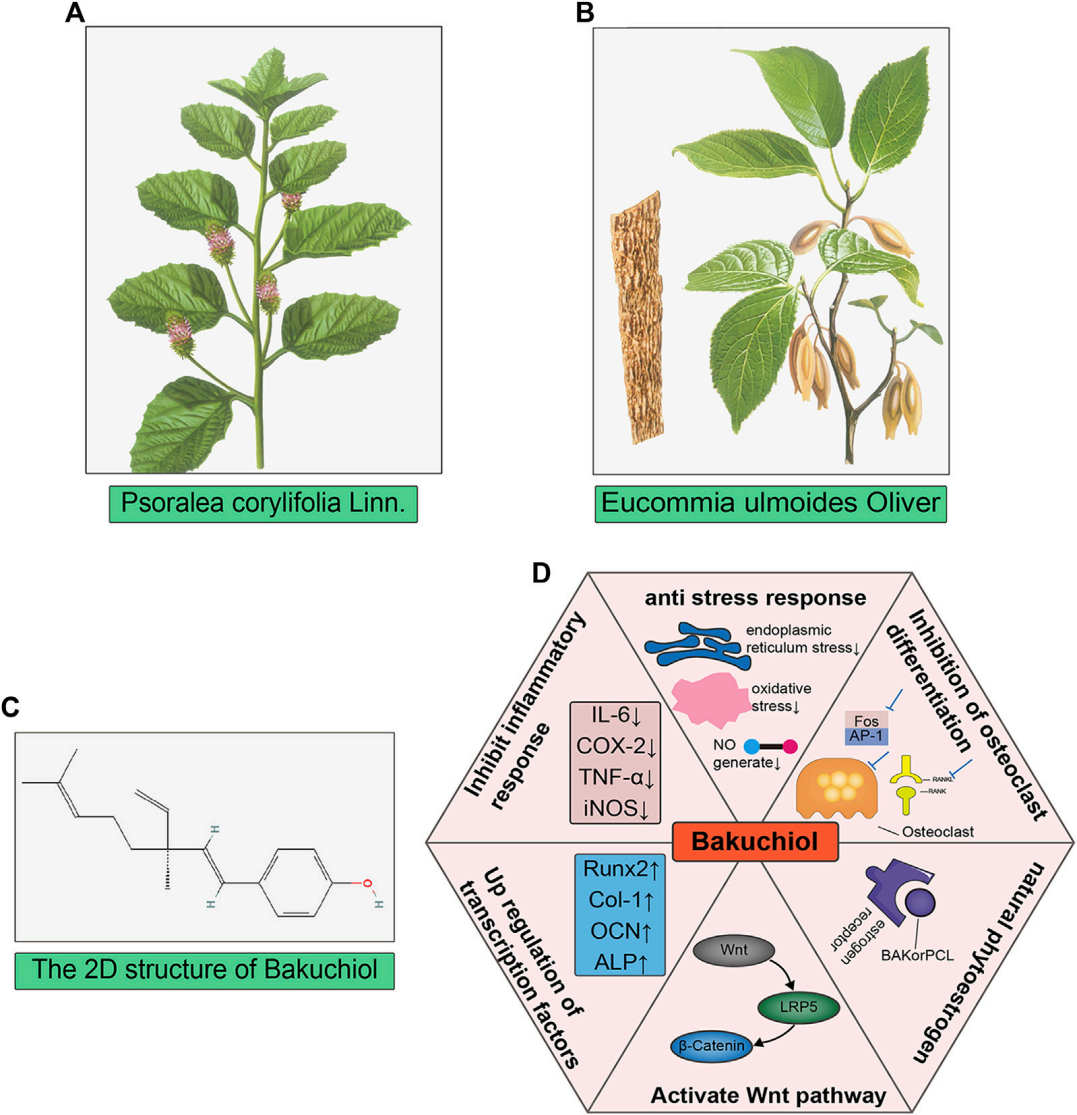
Mechanism diagram of RANKL/RANK/OPG signaling pathway cascade in osteoclastogenesis. Binding of RANKL to RANK leads to TRAF6 recruitment and thus activation of NF-kB and MAPK pathways. TRAF6 recruitment activates the NF-kB and JNK/ERK/p38 MAPK signaling pathways, and activation of the transcription factor Fos/AP-1 is also dependent on TRAF6 recruitment and JNK/ERK activation, together, these activated signals lead to activation of NFATc1, a key transcription factor for downstream osteoclast formation. In addition, RANKL activation stimulates the protein DAP12 or FcRγ of the tyrosine-activating motif ITAM, forming a complex of phospholipase C-γ and dephosphorylated tyrosinase, which activates calcium ion signaling and induces NFATc1 translocation into the nucleus of osteoclasts, and again here, NFATc1 works together with other transcription factors to induce osteoclast activation, thus NFATc1 is a marker event for osteoclast formation. The osteoclastogenesis inhibitory factor competitively binds RANKL and inhibits osteoclast differentiation thereby reducing osteoclast production.

For example, the compound bakuchiol isolated from Psoralea corylifolia Linn (PCL), PCL, and Modified Qing’ E Formula (MQEF) with PCL as the monarch drug, both of them can show promising anti-osteoporosis effect. Bakuchiol (BAK) is a compound isolated from PCL that inhibits M-CSF and RANKL co-stimulation-induced osteoclast differentiation and bone resorption via AKT and AP-1 pathways (Chai et al., 2018). In addition, BAK also acts on osteoblasts to induce osteoclast differentiation through upregulation of transcription factors Runx2, Collagen-I, ALP, osteocalcin (OCN) and activation of Wnt3a, LRP5 and β-catenin on the Wnt pathway (Weng et al., 2015). Furthermore, BAK is a natural estrogen receptor α (ERα) agonist that reduces postmenopausal bone loss by increasing ALP, calcium ion concentration, and serum estradiol concentration (S. H. Lim et al., 2009). BAK also has good anti-inflammatory and antioxidant effects and inhibits LPS stimulation-induced elevation of iNOS, COX-2, and TNF-a by inhibiting the p38 MAPK/ERK signaling pathway (H. S. Lim, Kim, Kim, & Jeong, 2019). In addition, BAK also has various effects such as inhibiting nitric oxide (NO) production and reducing oxidative stress damage (Liu et al., 2020; Xu, Lv, Liu, Zhang, & Yang, 2021). Inflammatory factor activation, NO production and oxidative stress damage are all important pathological changes of OP, but there is a lack of relevant studies and further exploration is needed in the future. The herb PCL also has the same effect of promoting osteoblast proliferative activity, improving BMD, reducing serum osteocalcin and promoting bone formation as BAK(W. D. Li et al., 2014; S. H. Lim et al., 2009). PCL attenuates pro-inflammatory cytokine expression (Pai et al., 2021). MQEF, which consists of PCL as the monarch drug, also improves bone biomechanics in osteoporotic ovariectomized mice by enhancing the expression of β-catenin on the Wnt pathway (Shuai et al., 2019). MQEF also increases the expression levels of adiponectin, BMP2 and OPG in patients with non-traumatic osteonecrosis, thus exerting a clinical protective effect (C. G. Li et al., 2017). In addition, MQEF was able to increase serum estradiol levels and reduce luteinizing hormone release, showing significant estrogenic activity in animals (Y. Xu et al., 2010) and the MQEF combination had more potent estrogen-like effects compared to the individual components, and the use of the formula alleviated the loss of appetite in animals caused by the use of PCL alone, which could explain the synergistic effect of MQEF with efficacy enhancing and toxicity reducing compared with PCL alone Figure 3: TCM for the treatment of osteoporosis (Xiong et al., 2022). In China, MQEF is recommended as a drug in the primary osteoporosis treatment guidelines, and the existence of MQEF-related Chinese patent drug also provides more convenience for patients. However, the lack of phase III clinical trials has prevented it from being used globally.

The screening of active ingredients from herbal medicines and herbal compounds, and studies of proximal mechanisms of action, specification of safety ranges by toxicological methods, standardized phase III clinical trials to determine efficacy and indications, and application of modern technologies to establish quality control and standardized testing of herbal medicines are required.

### Novel Biological Therapeutics Targeted to OP

#### RANKL Inhibitors

**FIGURE 4:**
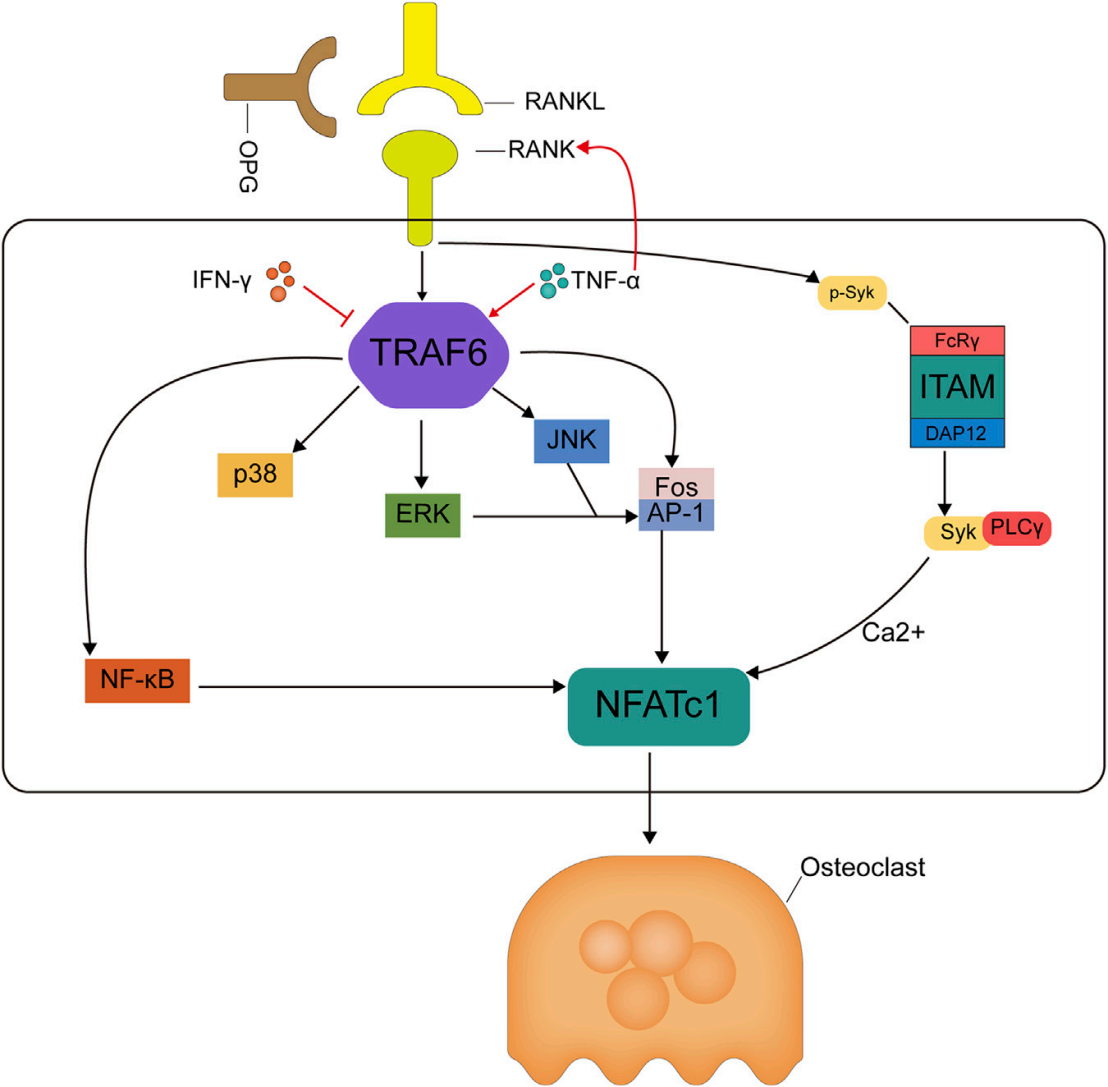
TCM for the treatment of osteoporosis. **(A,B)** The image of *Psoralea corylifolia Linn* and *Eucommia ulmoides Oliver*, both of them are the monarch drugs of Qing’ E formula; **(C)** The 2D chemical structure of bakuchiol, which is one of the main components in *Psoralea corylifolia Linn.* **(D)** showed the six main mechanisms that bakuchiol exerts anti-osteoporosis.

RANKL was initially identified as a novel member of the tumor necrosis factor receptor (TNFR) family expressed on dendritic cells and involved in dendritic cell-mediated T cell proliferation and the activation of RANK + T cells (D. M. Anderson et al., 1997). RANKL and bone metabolism have been studied extensively owing to the important role of the RANK/RANKL signaling pathway in osteoclast formation (Hsu et al., 1999; McDonald et al., 2021). RANKL, a homotrimeric transmembrane protein, exists as three isoforms. RANKL-1 and RANKL-2 have transmembrane structural domains, but RANKL-2 has a shorter intracellular structural domain and RANKL-3 has no transmembrane structural domain and is therefore soluble (Ikeda, Kasai, Utsuyama, & Hirokawa, 2001). Nuclear factor of activated T cells c1 (NFATc1) is the most potent transcription factor induced by RANKL and is essential for osteoclast activation, and activation of the calcium-NFATc1 pathway is essential for osteoclastogenesis and differentiation (Ikeda et al., 2001; Negishi-Koga & Takayanagi, 2009). RANKL induces *NFATc1* gene expression primarily by various mechanisms. RANK activates NF-κB by interacting with TNF receptor-associated factors (TRAFs). RANK contains two independent TRAF binding domains: the TRAF1, 2, 3, 5, and 6 binding regions (amino acids 544–616) and the TRAF6 binding region (amino acids 340–421). RANK-mediated NF-κB signaling is completely inhibited by the deletion of the TRAF6-binding region, indicating that the RANK-TRAF interaction is required for RANK activation (Jules et al., 2015). RANK-specific activation mediated by TRAF6 leads to the activation of NFATc1, a key transcription factor for osteoclast differentiation. In addition, in conjunction with RANK and co-stimulatory receptors, immunoreceptor tyrosine-based activation motif (ITAM) is phosphorylated by tyrosine kinase, leading to the activation of spleen tyrosine kinase (Syk) and phospholipase Cγ (PLCγ) (Koga et al., 2004). This forms an osteoclastogenic signaling complex, in turn activating calcium ion signaling, which is required for the induction and activation of NFATc1. c-fms is a receptor for macrophage colony-stimulating factor (M-CSF), and their binding leads to the upregulation of c-Fos, a component of the AP-1 transcription factor dimer complex, which is essential for osteoclast differentiation (Feng & Teitelbaum, 2013). c-Fos upregulation leads to the expression of RANKL and its receptor RANK, and the loss of c-Fos leads to a complete lack of osteoclast differentiation and the development of severe osteopetrosis (Wagner & Eferl, 2005). The AP-1 complex also plays a major role in NFATc1 amplification (Takayanagi et al., 2002). Therefore, inhibiting osteoclast production and differentiation by inhibiting RANKL activity, thus inhibiting bone resorption, may be an efficient strategy for the treatment of OP (Figure 4: Mechanism diagram of RANKL/RANK/OPG signaling pathway cascade in osteoclastogenesis). A clinical study of denosumab, an anti-RANKL monoclonal antibody, in postmenopausal patients with OP showed that 12 months of treatment resulted in a lower risk of fracture and lower incidence of reported adverse reactions, such as osteoarthritis, than those in the placebo control group (Cosman et al., 2016). Ten years of denosumab treatment had even greater benefits, including a sustained reduction in the fracture incidence and sustained increases in BMD (Bone et al., 2017). Based on the significant loss of antiresorptive effects within 7 months after a single injection of denosumab, which may lead to an increased risk of rebound-associated vertebral fractures, injections must be administered every 6 months (Lyu et al., 2020).

#### Romosozumab

Sclerostin, encoded by the *SOST* gene and secreted by osteoblasts, inhibits osteoblast differentiation and reduces osteogenesis. In mice, sclerostin (Tg-Sost) overexpression inhibits osteoblast activity, increases osteoclast proliferation, decreases serum 1a, 25-hydroxyvitamin D concentrations, increases FGF23, and decreases calcium and phosphorus levels (Winkler et al., 2003), whereas Sost knockout (Sost^−/−^) mice exhibit the opposite phenotypes, with an increased relative polysaccharide content in bone, decreased organic salt maturation and crystallization, and decreased bone matrix mineralization (X. Li et al., 2008; Sebastian & Loots, 2018). Thus, sclerostin, the product of the *Sost* gene, is a key negative regulator of bone formation, and Sost inhibitors are expected to be effective targets for the treatment of OP (Dai et al., 2021). Sclerostin antagonizes Wnt signaling in osteoblasts (Kim et al., 2020). Activation of the Wnt pathway not only induces osteogenic differentiation of MSCs and the expression of osteoprotegerin by osteoblasts to inhibit bone resorption by osteoclasts but also acts directly on osteoclasts to inhibit their differentiation (van Bezooijen et al., 2007). Sclerostin promotes β-catenin phosphorylation and proteasomal degradation by binding to the nuclear Wnt receptor and also inhibits low-density lipoprotein receptor-related proteins 5 and 6 (LRP5/6) binding to DKK by binding competitively to LRP5/6, together antagonizing canonical Wnt signaling activation to promote osteogenesis (Little, Recker, & Johnson, 2002; Mao et al., 2002; Clevers, 2006; Bhat et al., 2007). Clinical studies have shown that romosozumab improves trabecular and cortical bone architecture and is more effective in increasing spine and hip BMD compared to teriparatide (McClung et al., 2014; Ishibashi et al., 2017). Romosozumab resulted in greater increases in lumbar, hip, and femoral neck BMD than those observed for denosumab (Miyauchi et al., 2019). However, romosozumab may carry a potential risk of cardiovascular mortality and stroke (Tankó et al., 2005; Saag et al., 2017), thus limiting its use in patients with OP and concomitant cardiovascular disease, in which the benefits and risks must be weighed to maximize the clinical benefit.

#### Odanacatib (Cathepsin K Inhibitor)

Cathepsins are a class of cysteine proteases that degrade proteins in a variety of tissues. Cathepsin K (Cat K) is highly expressed in osteoclasts and is primarily responsible for the degradation of type I collagen. The inhibition of cathepsin K activity effectively inhibits bone resorption, as inorganic bone minerals are primarily degraded by the release of acidic ions, whereas organic bone matrix is primarily degraded by cathepsin K (Dodds et al., 2001). In mice with *CatK* gene knockout, osteoclasts have normal survival but bone resorption is inhibited (Gentile et al., 2014). In contrast, *CatK* transgenic mice exhibit increased bone resorption and bone turnover (Saftig et al., 1998; Kiviranta et al., 2001; Leung, Pickarski, Zhuo, Masarachia, & Duong, 2011); these processes are reversible (Zhuo, Gauthier, Black, Percival, & Duong, 2014). Based on these studies, odanacatib was developed and entered in a phase III clinical trial, demonstrating better or similar effects in increasing BMD and reducing fracture risk compared to those of denosumab (Harris et al., 1999; Black et al., 2007). Adverse events include cerebrovascular events, which may be related to molecular and cellular alterations in the central nervous system caused by low levels of cathepsin K in response to odanacatib, thereby affecting tissue homeostasis (Dauth et al., 2011; Drake, Clarke, Oursler, & Khosla, 2017).

In addition to these targeted anti-OP drugs, other targeted drugs are in development and in pre-clinical trials, such as the Src inhibitor saracatinib and the anti-DKK-1 antibody BHQ-880. Given the important role of these targets in OP, these drugs have great potential for clinical use and offer an encouraging strategy for the treatment of OP in the future (Heath et al., 2009; Rachner, Khosla, & Hofbauer, 2011).

## Discussion

Recent advances in our understanding of bone biology have revealed new options and have prompted unprecedented progress in the treatment of OP. The clinical use of denosumab and romosozumab is a good example. In this review, we provide an overview of commonly used anti-OP drugs, their pharmacological and molecular mechanisms, and promising targeted anti-OP therapeutics.

Bone remodeling is mediated by both osteoblasts and osteoclasts. Osteoblast differentiation is regulated by three key transcription factors, Runx2, Osterix, and β-catenin, which are essential for the induction of the differentiation of bone marrow MSCs into osteoblasts (Koga et al., 2005; Long, 2011). Osteoclast regulation is affected by the M-CSF/c-Fos and RANKL/RANK/OPG pathways. The Wnt pathway is the most important in the regulation of bone remodeling and bone metabolism (Gori, Superti-Furga, & Baron, 2016), and members of the Wnt family are indispensable for the processes of osteogenic differentiation, bone metabolism balance, and bone remodeling. DKK-1 secreted by osteoblasts binds to the BP1 and BP3 structural domains of LRP5/6 receptors in the Wnt signaling pathway, and secreted SOST binds to the BP1 structural domain of LRP5/6 and LRP4 receptors in the Wnt signaling pathway (Patel & Karsenty, 2002). This competitive binding enhances the inhibitory effects on the canonical Wnt pathway, and the knockout of Dkk1 or Sost significantly increases the bone content. The canonical Wnt pathway induces osteogenic differentiation via β-catenin by increasing the expression of target genes, including *Runx2, Osterix,* and *OPG*, increasing OPG protein expression, and inhibiting RANKL/RANK interactions to inhibit bone resorption (Baron & Kneissel, 2013). The non-canonical Wnt pathway regulates osteogenesis and bone resorption by the regulation of Wnt ligands, such as Wnt5a, the most strongly expressed of all Wnt ligands, and activates non-canonical Wnt signaling via the receptor tyrosine kinase-like orphan receptor (Ror) protein. Wnt5a is expressed by cells in the osteoblast lineage, whereas Ror2 is expressed by osteoclast precursors. The knockout of Wnt5a in osteoblasts or knockout of Ror2 in osteoclasts leads to reduced osteoclast formation and inhibits Wnt5a-induced osteoblast differentiation. Wnt5a ligands bind to Wnt receptors, leading to the activation of the Ror2 signaling cascade, which in turn activates the JNK/c-Jun pathway and enhances RANKL/RANK-induced osteoclastogenesis (Maeda et al., 2012; Hasegawa et al., 2018). Similar to conventional anti-OP therapeutics, novel targeted anti-OP drugs exert anti-OP effects by regulating the RANKL/RANK/OPG axis and the Wnt pathway. However, unlike the broad-spectrum targeting effects of conventional anti-OP drugs, targeted anti-OP drugs bind specifically and selectively to a receptor to affect osteoblast and osteoclast functions in bone remodeling. Despite their promise, some issues in the clinical use of targeted drugs remain, such as difficulties in development, high prices, and the presence of side effects that potentially outweigh the benefits. Therefore, conventional anti-OP drugs cannot be ignored and novel, effective, targeted anti-OP drugs with fewer side effects should be developed. Clinical observation studies and systematic evaluations and meta-analyses are needed to help these drugs reach the clinic more quickly. For conventional anti-OP drugs, in-depth research on their molecular mechanisms and pharmacological effects should be conducted along with toxicological and safety studies to guide the rational use of these drugs, eliminate drugs with few benefits and high risks, and improve and update our conceptual understanding of these drugs.

The safety of combinations of anti-OP drugs is also a concern. Different anti-OP drugs have different mechanisms of action, and the combination of drugs that inhibit bone resorption and drugs that promote bone formation may be an effective strategy for the treatment of OP. Some combinations may be effective, such as the combination of teriparatide and denosumab or zoledronate to increase BMD, whereas other combinations either show lower efficacies than those of single agents or lack sufficient clinical evidence (Eastell & Walsh, 2013; Tsai et al., 2013). It may be reasonable to consider the sequential use of drugs, which avoids the increased risk of specific diseases and minimizes resistance and peak effects caused by the long-term use of a single drug. Drug safety is directly related to patient safety issues, and some anti-OP drugs have been restricted or even withdrawn owing to inherent defects or clinical risks that far exceed the benefits. For example, fluoride ions are rarely used in conventional anti-OP therapies owing to the high incidence of gastrointestinal reactions and a tendency to cause fluorosis. The risk of heart disease and venous thrombosis associated with strontium salts is gradually being recognized and their use is decreasing. Novel anti-OP targets are rapidly moving into the clinic; however, their adverse events are unclear, which poses a challenge for drug safety studies.

In summary, anti-OP drugs are developing rapidly, and a comprehensive understanding of OP therapeutic agents and their molecular mechanisms of action is necessary. This review introduces anti-OP drug therapy and its pharmacological mechanisms from four major categories and 14 subcategories. We hope to help clinicians and researchers understand the past and future of anti-OP drugs, update their concepts and keep up with the frontier to achieve standardized clinical use, pharmacological and toxicological studies of drugs and the development of new drugs. Only through joint efforts can we gain an advantage in the fight against OP, a potentially devastating disease of aging.
